# TnaA, a trithorax group protein, modulates *wingless* expression in different regions of the *Drosophila* wing imaginal disc

**DOI:** 10.1038/s41598-023-42169-z

**Published:** 2023-09-13

**Authors:** Marco Rosales-Vega, Diana Reséndez-Pérez, Mario Zurita, Martha Vázquez

**Affiliations:** 1 Departamento de Genética del Desarrollo y Fisiología Molecular, Instituto de Biotecnología, Universidad Nacional Autónoma de México, 62210 Cuernavaca, Morelos Mexico; 2 Departamento de Inmunología y Virología, Facultad de Ciencias Biológicas, Universidad Autónoma de Nuevo León, San Nicolás de los Garza, Nuevo León Mexico

**Keywords:** Developmental biology, Genetics, Molecular biology

## Abstract

*wingless* expression is exquisitely regulated by different factors and enhancers in the imaginal wing discs of *Drosophila melanogaster* in four domains: the dorsal band, the dorso-ventral boundary, and the inner and outer ring domains. *tonalli* is a trithorax group gene that encodes a putative SUMO E3 ligase that binds to chromatin to regulate the expression of its targets, including the *Hox* genes. However, its role in modulating gene expression is barely known. Here, we show that TnaA modulates the *wingless* expression at two domains of the wing disc, the dorso-ventral boundary and the inner ring. At first, *tonalli* interacts genetically with *Notch* to form the wing margin. In the inner ring domain, TnaA modulates *wingless* transcription. When the dosage of TnaA increases in or near the inner ring since early larval stages, this domain expands with a rapid increase in *wingless* expression. TnaA occupies the *wingless Inner Ring Enhancer* at the wing disc, meanwhile it does not affect *wingless* expression directed by the *Ventral Disc Enhancer* in leg discs, suggesting that TnaA acts as a *wingless* enhancer-specific factor. We describe for the first time the presence of TnaA at the *Inner Ring Enhancer* as a specific regulator of *wingless* in the development of wing boundaries.

## Introduction

Gene expression is exquisitely regulated in the wing disc by the Notch, Wingless, Hedgehog, and Decapentaplegic pathways, among others, to form an adult organ. Genes responding to these inputs direct tissue patterning through differentiation, proliferation, and cell death. The Notch and Wingless signaling pathways are highly conserved in metazoans. Developmental processes in which Notch is involved include lateral inhibition, lineage decisions, and boundary formation (reviewed in^1^), while Wingless works on balancing cell proliferation, cell fate specification, changes in polarity, and differential cell adhesion (reviewed in^2^).

The formation of boundaries requires Notch and Wingless signaling and is well studied at the *Drosophila* wing disc. The genes that encode the transcription factors of these pathways have, in turn, complex regulatory regions that differentially respond according to the position of the cell in the wing imaginal disc and consequently activate the appropriate developmental programs to give rise to the adult wing.

Notch signaling defines the identity of dorsoventral (D/V) boundary cells and is required for the localized expression of genes involved in the formation and patterning of the wing margin, such as *wingless* (*wg*), *cut* (*ct*), and *vestigial* (*vg*)^3–5^. The Notch pathway is activated through a cell–cell signaling process between the Notch receptor and its ligand of the DSL family (Delta, Serrate, Lag-2), leading to the nuclear import of the Notch Intracellular Domain (NICD). In the nucleus, there are complexes with the DNA-binding protein Suppressor of Hairless [Su(H)], also known as CSL [named after CBF1, Su(H), and Lag-1], to activate or repress target gene transcription^1,6^.

**Figure 1:**
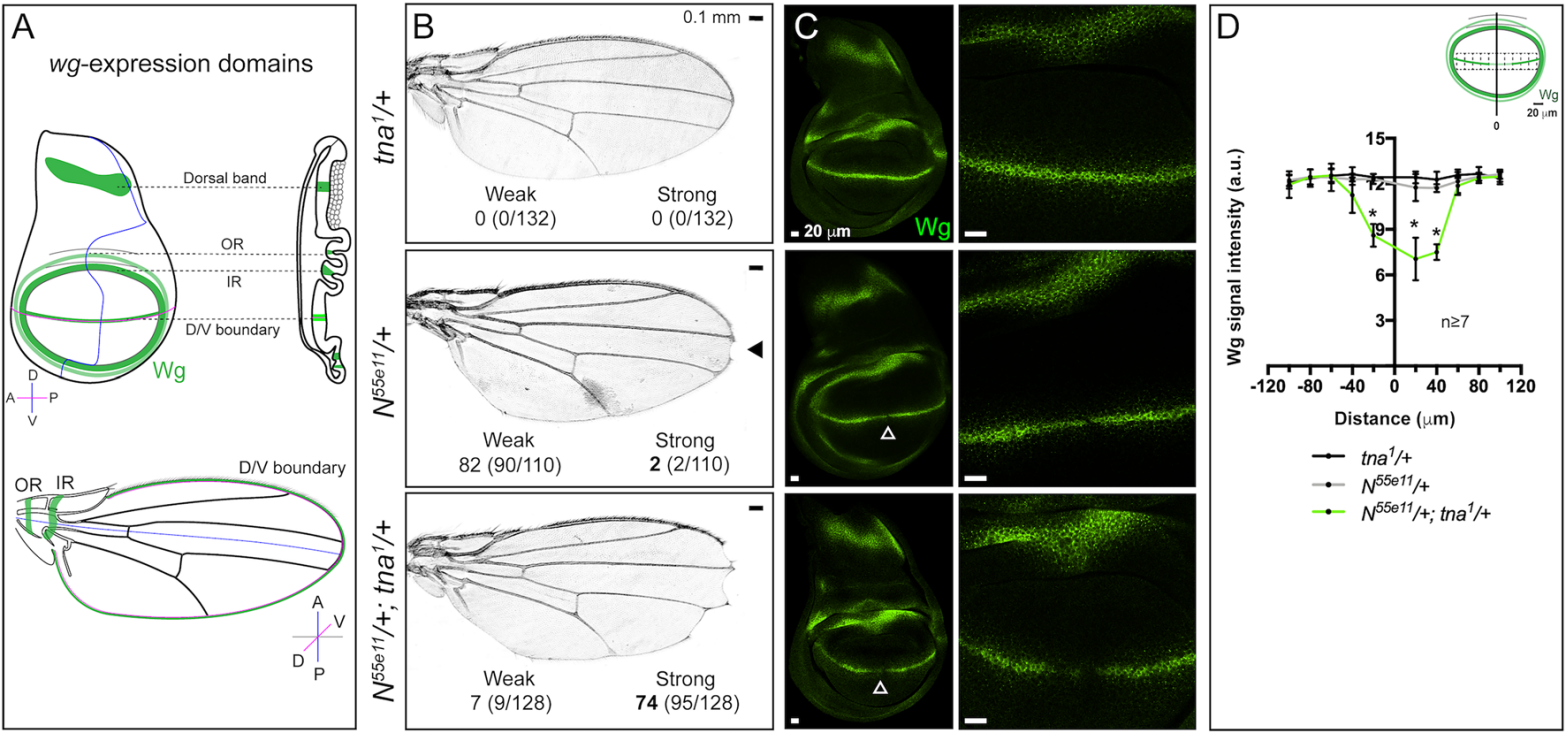
Genetic interaction of *tna* with *Notch* and *wg* expression in the wing disc. (**A**) Schemes of *wg* expression domains in the wing disc (green, upper panel) and their developmental fate in an adult wing (bottom panel). (**B**) Adult wings with *N* and/or *tna* mutant genotypes. Penetrance and expressivity of the notched-wing phenotype are classified as weak or strong when a single or several notches, respectively, are present in a wing margin. Quantification of each class is shown in Table 1. (**C**) Wingless signal in wing discs of *N* and/or *tna* mutant genotypes. Note that Wingless is further reduced at the D/V boundary domain in *N*^*55e11*^*/*+*; tna*^*1*^/+ wing discs (solid arrowhead, magnified in the right panels). (**D**) Quantification of Wingless in wing discs of the indicated genotypes (see Sup. Fig. 4B). Student’s *t*-test was performed for signal intensity in each bin (P < 0.05*).

Different regulators control the expression of *wg* to define the boundaries and domains on the wing disc^7,8^. In the late third instar wing disc, *wg* is expressed in a broad band located at the notum, along the D/V boundary, and in two concentric ring-like patterns at the hinge region called the inner (IR) and the outer (OR) ring domains^9^ (Fig. 1A). The D/V boundary domain will contribute to the formation of the adult wing margin, while the IR is necessary for the formation of the hinge^5^.

The Notch pathway regulates the expression of *wg* at the D/V boundary^4,5^, while Rotund and Nubbin are two key players in the regulation of *wg* expression in the IR domain^7^. *wg* has several known enhancers and at least two of them are differentially expressed in the imaginal discs. The *Inner Ring Enhancer* (*IRE*) acts on the wing disc^10^, while the *Ventral Disc Enhancer* (*VDE*) functions on the leg and eye-antennal discs^8^. However, the precise mechanisms that activate these enhancers and the factors that regulate their functions are poorly understood.

SUMOylation is a post-translational modification that modify the activity, location, and/or interaction of nuclear target proteins (reviewed in^11^). SUMO ligases favor the SUMOylation of specific target proteins. *tonalli* (*tna*) is a trithorax-group gene that is essential during larval and pupal development^12,13^. It encodes several TnaA isoforms with putative E3 SUMO ligase activity in the subunits of the BRAHMA complex, Osa and Brahma^13,14^ and probably in other nuclear proteins in imaginal discs.

In this work, we show that TnaA influences *wg* expression in the wing imaginal disc. We found that *tna* genetically interacts with *Notch* (*N*) to regulate *wg* expression at the D/V boundary domain. In the IR domain, we show through genetic and ChIP-qPCR experiments that TnaA favors *wg* expression since early larval stages and that it physically interacts with the *IRE*. In summary, the experiments presented here reveal TnaA as a positive modulator of *wg* expression, and its physical presence at the *IRE* describes for the first time a protein that may contribute to the function of this complex regulatory element.

## Results

### *tonalli* genetically interacts with *Notch* at the D/V boundary wing disc to form the wing margin

The wing disc is divided into different regions according to complex gene expression patterns. One of the genes that is expressed in these regions is *wg* (Fig. 1A).

In previous work, we identified modifiers of the BRAHMA complex that include *tna*^12^. Individuals with mutations in some of these BRAHMA modifiers, which did not include *tna* at that time, have notched wings among other phenotypes^15^. This notched wing phenotype resembles that exhibited by individuals with defects in the Notch signaling pathway at the D/V boundary of the wing disc (Fig. 1B).

To investigate whether *tna* is related to Notch signaling, we assayed the genetic interaction of *N* with *tna*, looking for the notched-wing phenotype in flies carrying the null *N* allele, *N*^*55e11*^, in combination with different *tna* alleles that include *tna*^*1*^, *tna*^*5*^, *tna*^*EY22929*^ and *tna*^*MI01482*^ (Fig. 1B, and Table 1, for location and description of the alleles, see Sup. Fig. 1A, and “Methods”). Female heterozygote individuals carrying any of the tested *tna* alleles have normal wings (e. g. *tna*^*1*^/+, Fig. 1B, Table 1). Most* N*^*55e11*^/+ females (82%) have wings with notches located at the distal tip of the wing blade (weak phenotype), while in the presence of *tna*^*1*^ (*N*^*55e11*^/+; *tna*^*1*^/ +), the flies show extensive notches located mainly along the posterior wing margin with strong (74%) or weak (7%) expressivity. Individuals with the other tested *tna* alleles also show the notched-wing phenotype with different penetrance and expressivity (Table 1). *N*^*55e11*^/+; *tna*^*1*^/+ mutant flies present a strong notched-wing phenotype, as the more evident loss-of-function phenotype of *N*, suggesting that TnaA is required for activities of the Notch pathway related to the formation of the wing dorso-ventral boundary.

**Table 1:** *tna* interacts genetically with *N* and *Su(H).*

Genotype^a^	Individuals with notched wings/total^b^	
	Weak	Strong
*tna*^*1*^*/*+	0/132 (0)	0/132 (0)
*tna*^*5*^*/*+	0/178 (0)	0/178 (0)
*tna*^*MI01482*^*/*+	0/125 (0)	0/125 (0)
*tna*^*EY22929*^*/*+	0/149 (0)	0/149 (0)
*N*^*55e11*^*/*+	90/110 (82)	2/110 (2)
*Su(H)*^*1*^	2/72 (3)	0/72 (0)
*N*^*55e11*^*/*+*; tna*^*1*^*/*+	9/128 (7)	95/128 (74)
*N*^*55e11*^*/*+*; tna*^*MI01482*^*/*+	13/129 (10)	55/129 (43)
*N*^*55e11*^*/*+*; tna*^*5*^*/*+	18/161 (11)	56/161 (35)
*N*^*55e11*^*/*+*; tna*^*EY22929*^*/*+	12/147 (8)	35/147 (24)
*Su(H)*^*1*^*/*+*; tna*^*1*^*/*+	15/91 (16)	0/91 (0)
*Su(H)*^*1*^*/*+*; tna*^*EY22929*^*/*+	7/86 (8)	0/86 (0)

^a^*N*^*55e11*^/*FM0* female virgins were crossed with males carrying different *tna* alleles.

^b^Number of individuals with the notched-wing phenotype. Penetrance and expressivity of the notched-wing phenotype are classified as weak or strong when a single or several notches, respectively, are present in a wing margin (see Fig. 1B). The percentages are indicated in parentheses. The results are a compilation of F1 of at least four independent crosses. Statistical significance in each case was determined with *x*^2^ (P < 0.05) as stated in “Methods”.

We also tested the genetic interaction between *tna* and *Su(H)* by combining the loss-of-function *Su(H)*^*1*^^16^ and the alleles *tna*^*1*^ and *tna*^*EY22929*^. We found that wing-notching in transheterozygote *Su(H)*/+*; tna*/+ individuals increases slightly with respect to notched wings of individuals carrying only the *Su(H)*^*1*^ allele (16% for *Su(H)*^*1*^/+*; tna*^*1*^/+, 8% for *Su(H)*^*1*^/+*; tna*^*EY22929*^/+ compared to 3% for *Su(H)*^*1*^*/*+ individuals) (Table 1 and Sup. Fig. 2A)^17^. Thus, there is a genetic interaction between *tna* and *Su(H)* although it is not as strong as the one found in *N*^*55e11*^/+*; tna*/+ individuals (Fig. 1B and Table 1).

Since *wg* expression is controlled by the Notch pathway at the D/V boundary^4,5^, we evaluated the Wingless protein level in wing discs of *N*^*55e11*^/+; *tna*^*1*^/+ flies. We found that the level of Wingless decreases, in correlation with the observed phenotypes in adult wings (Fig. 1C). Although the Wingless level at the D/V boundary is intact in discs from *tna*^*1*^/+ individuals, it is reduced in a few cells at the center of the D/V boundary in discs of *N*^*55e11*^/+ genotype. The latter phenotype is enhanced in *N*^*55e11*^/+; *tna*^*1*^/+ animals (Fig. 1C) and in all combinations of *tna* alleles tested (Table 1). Quantification of this phenotype is presented in Fig. 1D and Sup. Fig. 4A.

**Figure 2:**
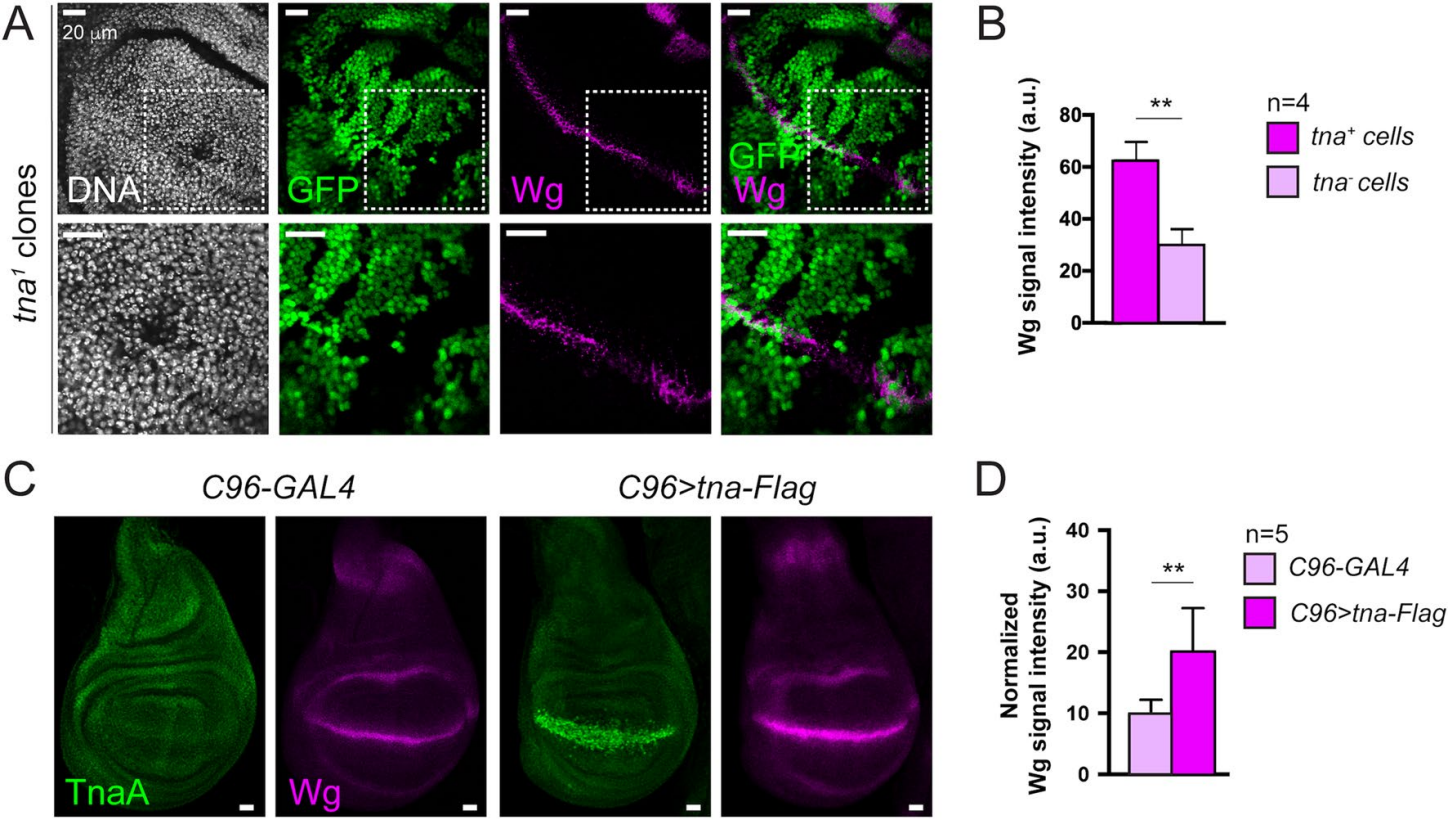
Wingless at the D/V boundary in *tna*-deficient and *tna-Flag* expressing wing discs. (**A**) Wingless signal in *tna*^*1*^ clones (non-GFP cells) induced in the D/V boundary with *Ubx-FLP*^46^. Dashed squares in the images in the upper panel indicate the amplified region in the lower panel. (**B**) Quantification of the intensity of the Wingless signal at the D/V boundary in *tna*^+^ and *tna*^*−*^ cells (**C**) Wingless signal in *tna-Flag* expressing discs at the D/V boundary driven by the *C96-GAL4* driver^59^. The discs were immunostained for TnaA and Wingless with the corresponding antibodies (“Methods”). (**D**) Quantification of Wingless in *tna*^+^ and *tna*^*−*^ cells as indicated in Sup. Fig. 4B. Student’s *t*-test was performed for the intensity of the signal in (**B**) and (**D**) (P < 0.01**).

To further test this finding, we asked whether the Wingless protein level decreased in *tna*^*1*^ clones (Sup. Fig. 3) that cross the D/V boundary since the expression of *wg* is controlled by NICD in this region^4,5^. In fact, we found that this is the case (Fig. 2A). Consistently, quantification of the Wingless signal is reduced almost 50% in these *tna*^*−*^ clones (Fig. 2B), and adult wings derived from these wing discs present notches (Sup Fig. 2B).

Next, we directed the expression of an epitope-tagged version of TnaA_123_ that we will name from now on TnaA-Flag, to the D/V boundary. As wild-type TnaA, TnaA-Flag is nuclear (Sup. Fig. 1B), binds to the same bands in polytene chromosomes (Sup. Fig. 1C), and, albeit partially, complements the lethality of *tna*^*−*^ individuals (see “Methods”). We found that in the presence of TnaA-Flag, Wingless show a two-fold increase along the D/V boundary (Fig. 2C,D and Sup. Fig. 4B), in contrast to the Wingless decrease found in this domain in *tna*^*−*^ clones (Fig. 2B), further supporting the notion that TnaA regulates *wg* expression.

**Figure 3:**
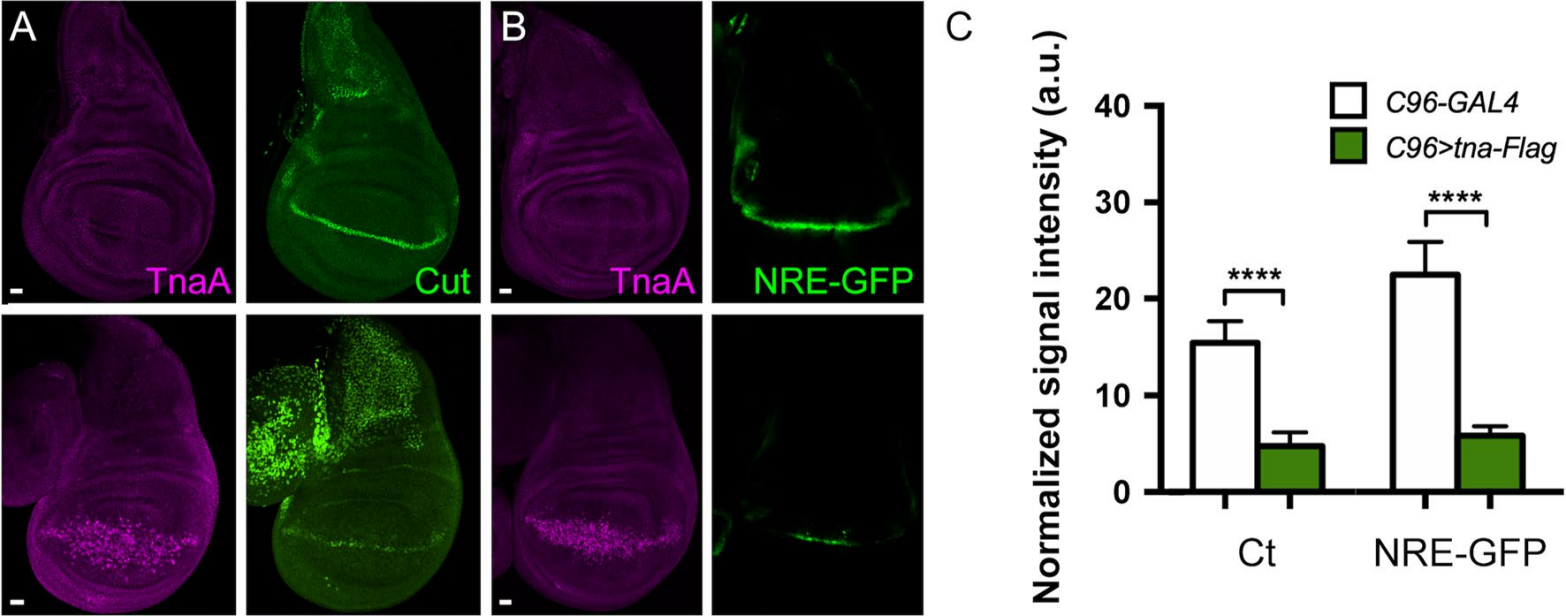
Cut and NRE-GFP signal in wing discs expressing *tna-Flag* along the D/V boundary. The expression of *tna-Flag* was driven to the D/V boundary with *C96-GAL4*^59^. (**A**) TnaA and Cut signals on *C96-GAL4* wing discs (upper panel), or in *C96* > *tna-Flag* wing discs (lower panel)*.* (**B**) TnaA and NRE-GFP signals in wing discs of genotypes as in (**A**). Note the reduction of Cut and NRE-GFP signals along the D/V boundary domain (**C**) Quantification of Cut and NRE-GFP in the D/V boundary of wing discs of the indicated genotypes (n ≥ 5 discs). See also Sup. Fig. 4B. Student’s *t*-test was performed for signal intensity in each case (P < 0.0001****).

We also analyzed the effect of TnaA-Flag presence at the D/V boundary on the levels of Cut whose expression is controlled by NICD in this region^5^ (Fig. 3A). We observed that Cut diminishes considerably at the D/V boundary of these wing discs (Fig. 3A,C, and Sup. Fig. 4B) that develop into defective adult wings (Sup. Fig. 2C). These results are similar to those observed by others in *Su(H)* mutant individuals^18,19^, suggesting a functional relationship between *tna* and *Su(H)*.

Next, we tested whether TnaA directly affects Notch-mediated transcriptional activation. We measured GFP expression in flies bearing a Notch pathway reporter construct *NRE*-*GFP* (*Notch Responsive Element*, *NRE*)^20^. We found that in the presence of TnaA-Flag, the expression of *NRE-GFP* is substantially reduced, indicating that TnaA interferes with the CSL complex-mediated activation of the *NRE-GFP* reporter in this specific region of the wing disc (Fig. 3B,C).

We conclude from the loss-of-function experiments that *tna* is interacting with the Notch pathway to control the level of Wingless at the D/V boundary domain of the wing disc. In addition, the positive and negative effects of TnaA-Flag on *wg* and *ct,* respectively, agree with the reported formation of Su(H)-CSL activating and repressor complexes on the promoters of these genes under *Su(H)* overexpression^18,19^ (see “Discussion”).

### TnaA modulates the expression of *wg* in the IR domain of the wing disc

We previously established that *tna* genetically interacts with the Notch pathway to control the level of Wingless protein at the D/V boundary. Next, we knocked-down TnaA (a decrease of approximately 70% with respect to the wild-type level), by expressing a *tna* RNAi (*tna*^*JF02536*^,^13,21^) in the anterior compartment of the wing disc (Sup. Fig. 5) and assessed Wingless levels in their different expression domains (IR and D/V boundary) in this compartment. In this otherwise wild-type background, we did not observe any Wingless fluctuation in any of the evaluated regions, compared to the correspondent regions at the posterior compartment that are expressing wild-type TnaA levels (Sup. Fig. 5).

We reasoned that the TnaA knockdown level reached by the expression of *tna*^*JF02536*^ RNAi may not be enough to knock down the robust Wingless expression in the different regions of the disc. We hypothesized that TnaA may act on a particular *wg*-regulatory element. *wg* has several embryonic and larval enhancers. One of the larval enhancers is the wing disc IR enhancer (*IRE*), located about 9 kb upstream the *wg* promoter^10^.

**Figure 4:**
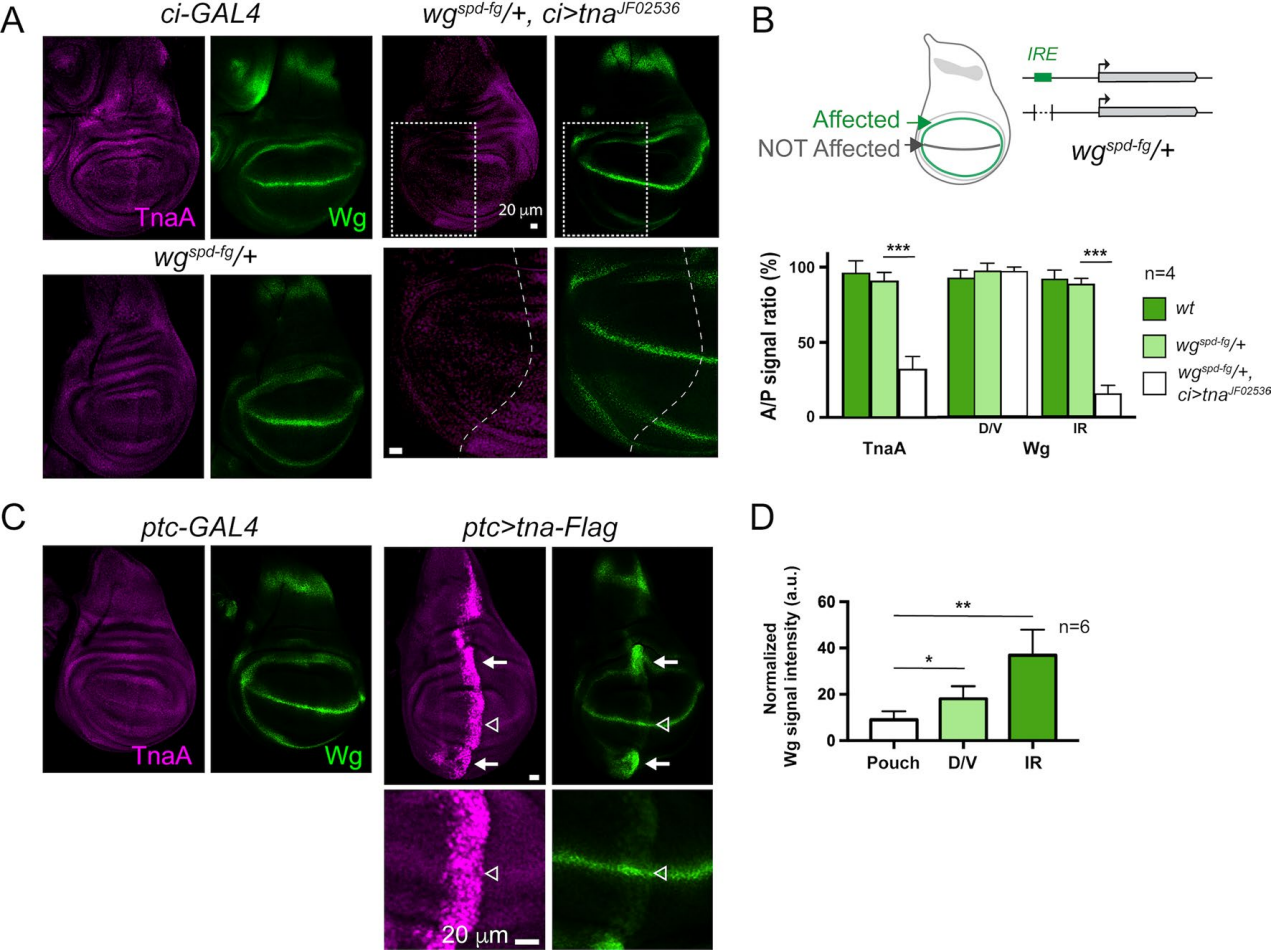
Effect of the dosage of TnaA on *wg* expression in the IR (**A**) The Wingless signal in the IR decreases when TnaA is knocked down in *wg*^*spd-fg*^/+ wing discs. Immunostainings of TnaA and Wingless in wing discs of the indicated genotypes. TnaA was knocked down in the anterior compartment with the expression of the *tna*^*JF25036*^ RNAi driven by the *ci-GAL4* in *wg*^*spd-fg*^*/*+ discs (*wg*^*spd-fg*^/+; *ci* > *RNAi-tna*^*JF02536*^). The dashed rectangles (right upper panels) show the amplified region (right lower panels) where the A/P margin is indicated. (**B**) The *wg*^*spd-fg*^ allele is a small deletion that removes the *IRE* region (upper diagram) and directs the expression of *wg* to the IR (green), but not to the D/V boundary (grey). Quantification of TnaA and Wingless A/P signal ratio in the D/V boundary and IR in discs with the indicated genotypes (lower panel). (**C**) TnaA and Wingless signals in wing discs that express *tna-Flag* at the anteroposterior margin driven by *ptc-GAL4*^60^ that is active since the early second instar stage^61^. The IR (white arrows) and the D/V boundary (empty arrowhead) regions on the A/P axis are indicated (**D**) Quantification of the Wingless signal in the pouch, D/V and IR in *ptc* > *tna-Flag* wing discs (n ≥ 6 discs). See also Sup. Figure 4C. Student’s *t*-test was performed for signal intensity in (**B)** and (**D**) in each case (P < 0.05*, P < 0.01**, P < 0.001***).

We chose to test the TnaA requirements for the function of this enhancer in an *IRE* sensitized background. We used a line that carries the *wg*^*spd-fg*^ allele, which is a small deletion that removes the *IRE* region. Wing discs where the deletion is homozygous lack *wg* expression at the IR while the one at the D/V boundary remains unchanged^10^. When the *IRE* is haploinsufficient, the Wingless signal looks normal in the IR and in the D/V (Fig. 4A, *wg*^*spd-fg*^/ +). The quantification of the Wingless and TnaA signals in each of these regions is shown in Fig. 4B. In contrast, when TnaA is knocked down in the anterior compartment of the wing disc (*wg*^*spd-fg*^/+, *ci* > *tna*^*JF02536*^), there is a decrease in the Wingless signal in the anterior half of the IR (Fig. 4A, *wg*^*spd-fg*^/+, *ci* > *tna*^*JF02536*^). An internal control of this experiment is the normal Wingless signal observed in the posterior IR region where *tna*^*JF02536*^ is not expressed. Moreover, in this *wg*^*spd-fg*^/+ background, Wingless is not reduced at the D/V boundary as expected, since the *IRE* does not control the *wg* expression in this domain. The quantification of the TnaA and Wingless levels in these discs is shown in Fig. 4B.

To complement these data, we directed *tna-Flag* expression to the anteroposterior (A/P) margin of the wing disc (Fig. 4C,D). In this case, we did observe TnaA-Flag effects on *wg* expression at both the D/V boundary and the IR (Fig. 4C**,** open and solid arrows, and quantification of the Wingless signal for each region in Fig. 4D and Sup. Fig. 4C). In response to TnaA-Flag, *wg* expression increases at the D/V boundary (Fig. 4C, open arrowhead). At the IR, *wg* expression is also increased, and its expression domain is expanded (Fig. 4C solid arrows). Adult wings derived from these discs show defects along the A/P boundary, including the hinge (Sup. Fig. 2D).

In conclusion of these experiments, in addition to influencing the expression of *wg* at the D/V boundary, TnaA also modulates its expression at the IR possibly through some direct or indirect action on the *IRE*.

### The presence of TnaA-Flag in various developmental stages induces a rapid expansion of the Wingless IR domain

**Figure 5:**
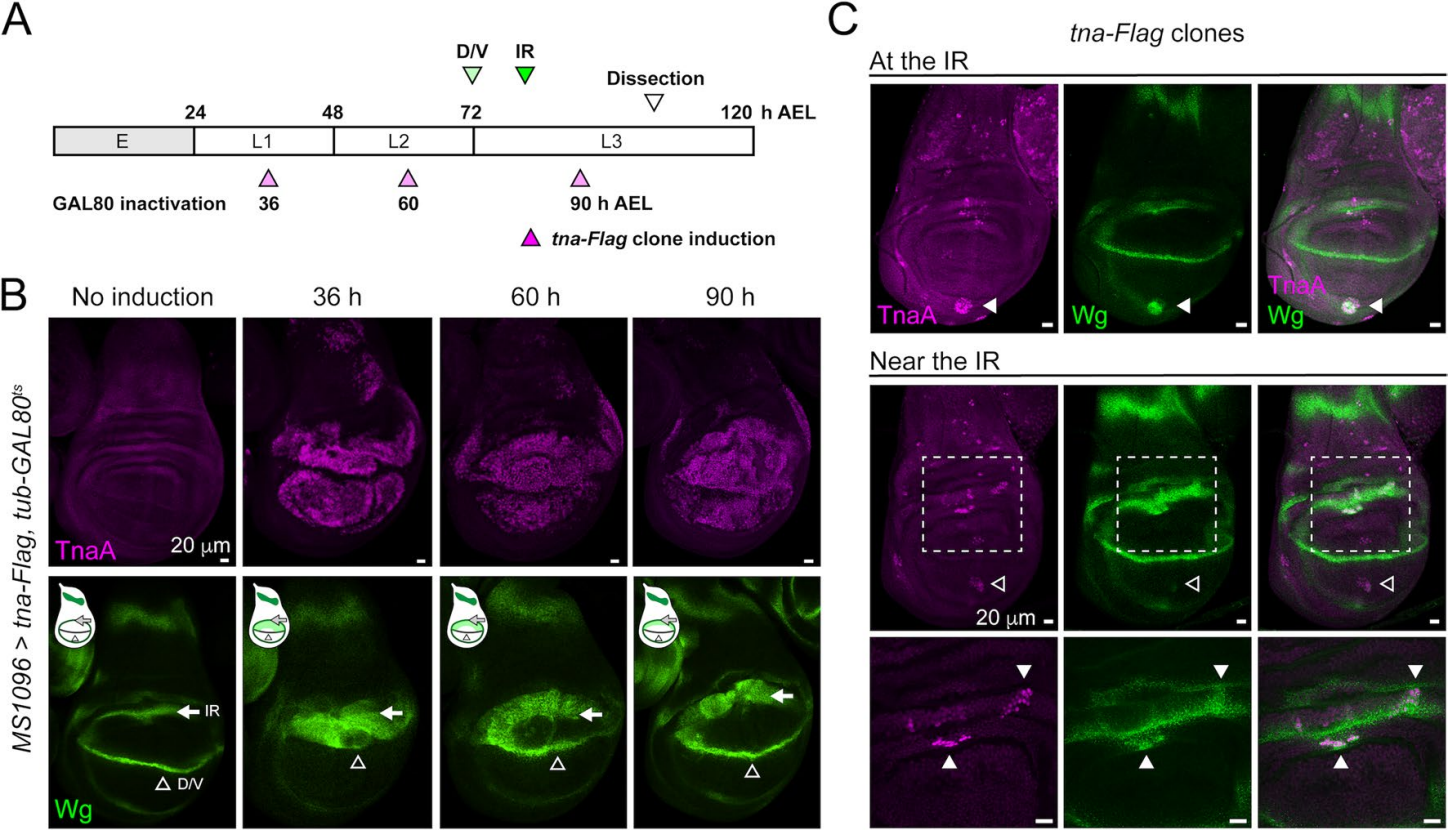
The TnaA-Flag effect depends on its timing of appearance and proximity to the IR during larval development. (**A**) Developmental timeline (E, Embryo, L1-L3 larval stages) according to hours after egg laying (h AEL). The time when the expression of *wg* is resolved in the D/V and IR domains (green arrowheads), and the temperature shift times applied to inactivate Gal80ts to allow expression of *MS1096* > *tna-Flag* at the dorsal hinge (pink arrowheads) are indicated. (**B**) TnaA and Wingless distribution in wing discs expressing *tna-Flag* at specific times from 36 to 90 h AEL. Note the expansion of the Wingless signal from the IR (white arrow) but not from the D/V boundary domain (empty arrowhead). (**C**) Expression of *wg* in TnaA-Flag clones in different regions of the wing disc. TnaA and Wingless immunostainings are indicated, and merged images are shown (right panels). Images from two discs show a TnaA-Flag clone at the IR (upper panel) or near the IR (middle and lower panels). A TnaA-Flag clone in the pouch does not cause an induction of *wg* expression (middle panel, empty arrowhead). TnaA-Flag clones at/or adjacent to the *wg* IR domain present the induction of *wg* expression (middle and lower panels, solid arrowheads). Dashed squares (middle panel) indicate the region amplified in the lower panel.

The characteristic pattern of *wg* expression in the third instar wing discs results from the activation of different enhancers along earlier larval stages. The larval expression of *wg* begins at the second instar (48 h after egg laying, AEL) in a ventral anterior region of the wing discs^26^ directed by a well characterized early enhancer^27^. In early third instar larvae, the expression of *wg* is detectable in the D/V boundary and the IR^7,27^ (Fig. 5A).

We investigated whether TnaA acts at a specific time of larval wing disc development. For this goal, we used the TARGET system, which inactivates the GAL4 repressor (GAL80ts) with temperature shifts^28^. We applied temperature shifts to inactivate GAL80ts at specific times along the development of the wing discs. This allowed us to accurately control the induction of *tna-Flag* expression in the *MS1096-GAL4* driver pattern between second and third instar larval stages (72 h AEL)^29,30^ (Fig. 5A, see “Methods”). We found that as a result of *tna-Flag* expression since the first instar larval stage (36 h AEL), there is a strong expansion of the IR towards the pouch **(**Fig. 5A,B**)**. At the third instar larval stage (90 h AEL), induction of *tna-Flag* expression causes a milder expansion of the IR domain than the one observed when induced at an earlier developmental stage (Fig. 5B, solid arrow).

We also studied whether cells in different regions of the wing disc could induce *wg* expression in response to TnaA dose. To approach this question, we induced clones expressing *tna-Flag* on the entire disc at 82 h AEL using the FLP-out technique (Fig. 5C and “Methods”). As previously shown, there is an expansion of the IR towards the pouch or the hinge when clones locate at/or near the IR (Fig. 5C, solid arrowheads). We also noticed that these cells rapidly increase the Wingless level in response to TnaA-Flag since the clones were induced only 24 h before dissection. In contrast, clones far from the IR, in other regions of the wing disc, such as the notum or ventral pouch (Fig. 5C empty arrowhead), do not show almost any increase in the level of Wingless. This indicates that proximity to the IR domain is important for the regulation of *wg* transcription mediated by TnaA.

These results show that TnaA-Flag can activate the expression of *wg* in the IR even before this domain is resolved in early to mid-third instar larvae. This TnaA-Flag effect is milder but is still observed when its expression is induced after the IR formation. Moreover, TnaA-Flag can rapidly increase *wg* expression, particularly in cells at or near the IR, with expansion of this domain. Altogether these results suggest that regulatory regions that modulate *wg* expression in the IR are available and highly sensitive to TnaA doses in specific stages of larval development.

### TnaA localizes at the IR enhancer to modulate *wg* transcription

**Figure 6:**
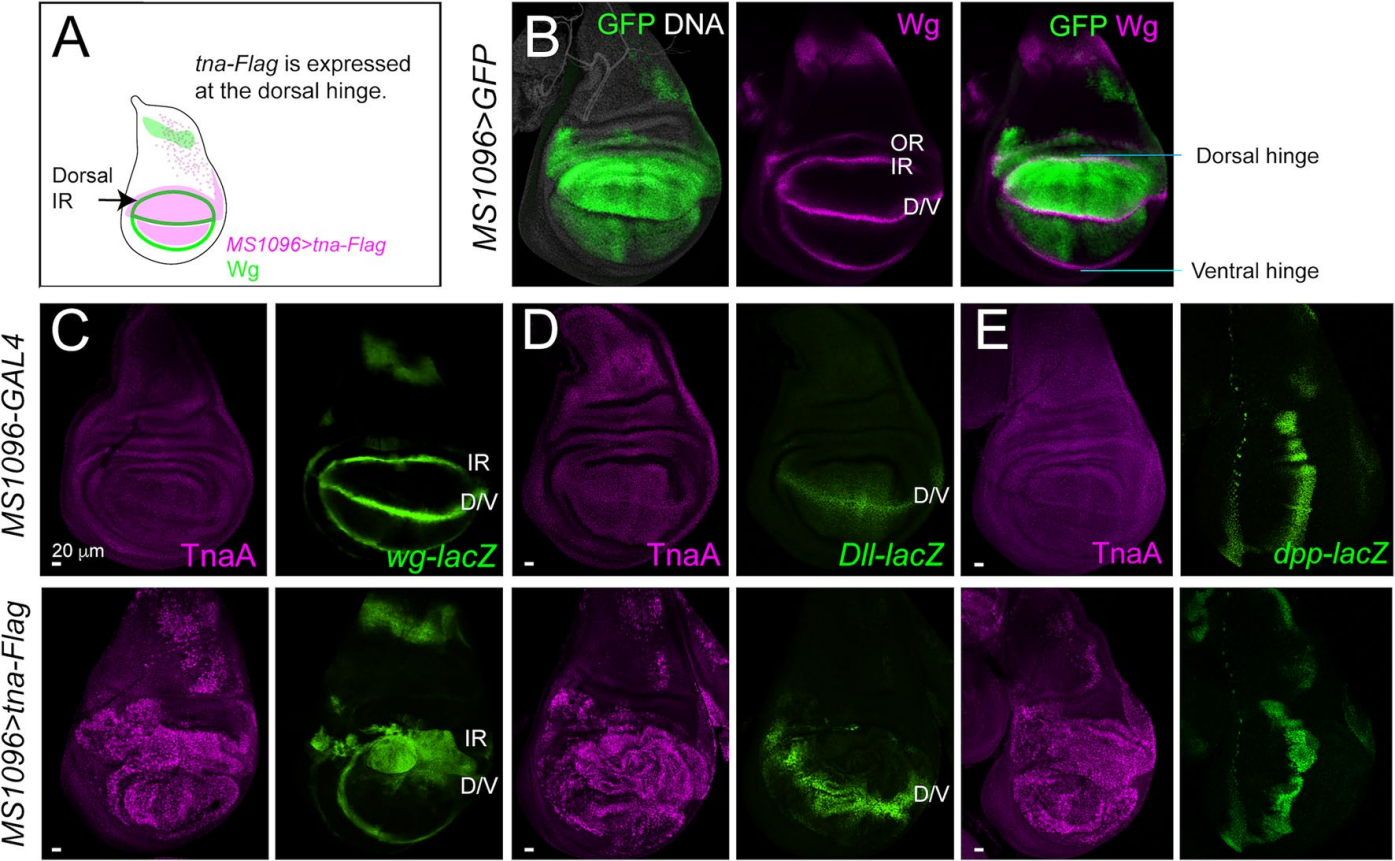
TnaA-Flag transcriptional effect on *wg* expression in the IR. The expression of *tna-Flag* was driven to the dorsal hinge with *MS1096-GAL4*^23^. (**A**) Scheme of the expression domain of *MS1096* > *tna-Flag* (magenta) and the *wg* expression pattern (green) in a wing imaginal disc. Under this condition, the expression of *tna-Flag* overlaps only with the *wg* IR dorsal side and not with the ventral side, which could be used as a control in the same disc. (**B**) *MS1096* > *GFP* wing discs showing GFP expression on the dorsal hinge (left), Wingless signal (middle) and merged image (right). (**C–E**) *MS1096-GAL4* and *MS1096* > *tna-Flag* wing discs, immunostained for TnaA (magenta) or LacZ (green). (**C**) Expression of the transcriptional reporter *wg-lacZ*^62^. Note that the ventral *wg* IR domain is not affected since the driver is not active in this region. (**D**) The D/V boundary followed by the *Dll-lacZ* reporter^25^. (**E**) The A/P boundary followed by the expression of *dpp-lacZ*^24^.

Next, we investigated whether TnaA directly affects *wg* transcription at the IR. We followed the expression of the *wg-lacZ* reporter^22^ at the IR in wing discs where *tna-Flag* expression was directed to the dorsal hinge with the *MS1096-GAL4* driver^23^. This allowed us to monitor the expansion of the IR domain on the dorsal side of the wing disc, leaving the ventral side as an internal control (Fig. 6A,B). We found that under this condition, the expression of *tna-Flag* increases *wg* transcription (Fig. 6C). This causes a strong expansion of the dorsal IR domain towards the pouch, making it hard to distinguish it from the D/V boundary. As *tna-Flag* is not expressed in this region, the ventral IR looks normal (Fig. 6C). We also corroborated that both the A/P and D/V boundary domains remained intact in these wing discs, by monitoring the expression of *dpp-lacZ*^24^ and *Dll-lacZ*^25^, respectively (Fig. 6D,E). These results reinforce the notion that TnaA specifically influences the transcription of *wg* at the IR domain.

**Figure 7:**
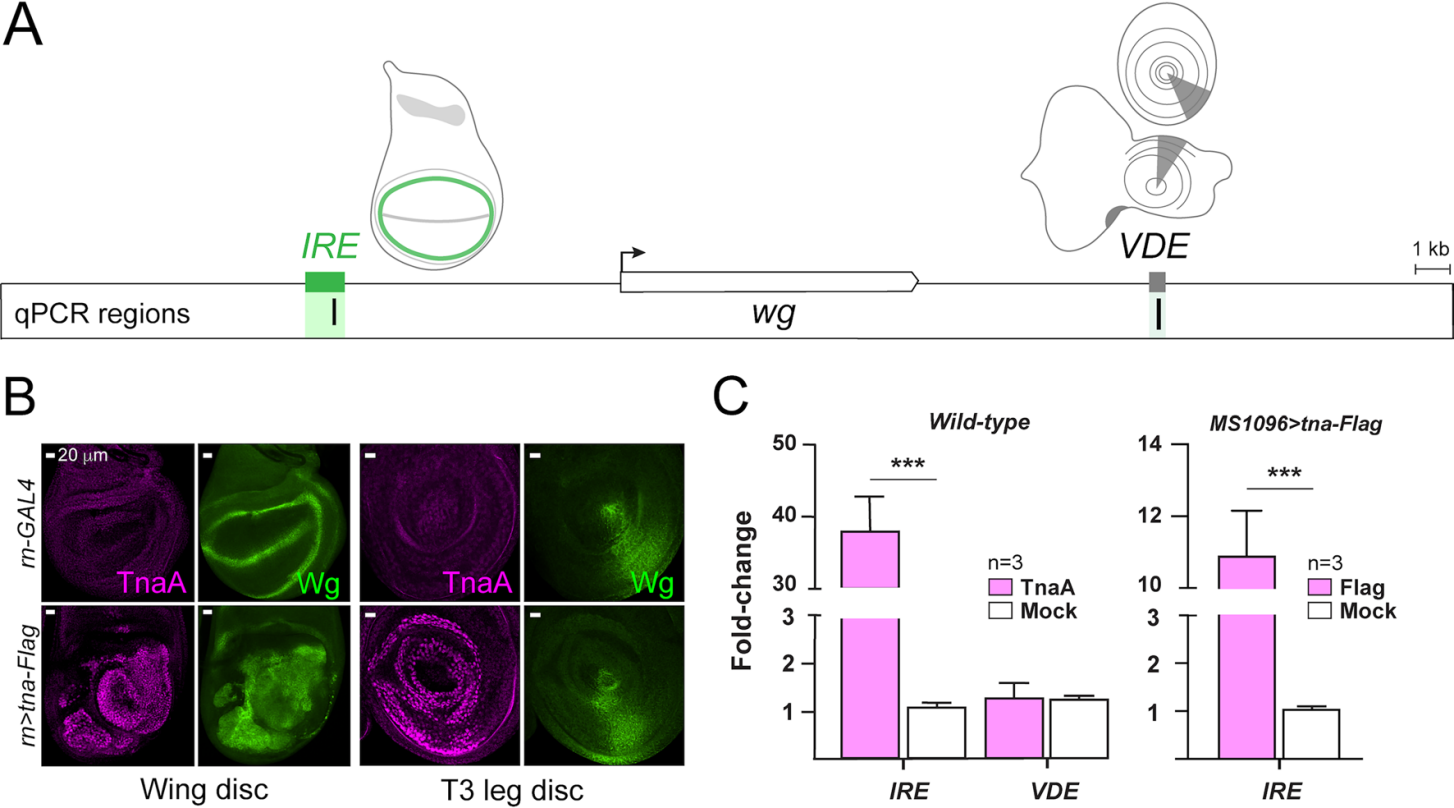
TnaA and TnaA-Flag are in the *IRE* in the wing disc. (**A**) Scheme of the *wg* locus showing the *IRE* and *VDE* that control *wg* expression in the wing and T3 leg discs (upper panel). The localization of the regions used (green lines) to evaluate the presence of TnaA by ChIP-qPCR with the anti-TnaA_TAIL_ antibody is indicated. (**B**) TnaA and Wingless immunostainings of the *rn-GAL4* and *rn* > *tna-Flag* wing and T3 leg discs (upper and lower panels, respectively). The expression of *tna-Flag* was directed to the distal part of the discs with *rn-GAL4* which is active since the late second instar stage^31^. Note that the TnaA-Flag wing disc shows expansion of the IR domain (green) that is correlating with the presence of TnaA in the *IRE* in the wing discs. In contrast, the morphology and the Wingless expression pattern are normal in the TnaA-Flag T3 leg disc, where the *IRE* is not active. (**C**) TnaA ChIP-qPCRs of the *wg IRE* and *VDE* in chromatin from wild type wing discs (left). The presence of TnaA-Flag in the *IRE* was determined by ChIP-qPCR with a FLAG antibody in *MS1096* > *tna-Flag* discs (right). qPCR reactions with immunoprecipitated DNA samples with anti-TnaA_TAIL_, anti-FLAG, or with irrelevant antibodies (rabbit and mouse IgG for TnaA and TnaA-Flag, respectively; see “Methods”) were performed with the indicated primer sets to amplify the selected *IRE* or *VDE* regions indicated in (**A**) (black vertical lines). Results are shown as fold enrichment over the background signals. Student’s *t*-test was performed on qPCR enrichments in the regions tested (P < 0.001***).

The *wg* locus has several enhancers that control its expression in different regions or stages of development. Imaginal *wg* enhancers include the *IRE* that controls *wg* expression in the IR domain of wing and haltere discs^10^, and the *VDE* which controls the antero-ventral *wg* expression in leg and eye-antennal discs^8^ (Fig. 7A). When the expression of TnaA is knocked down, the Wingless signal decreases in the IR in wing discs that harbor only one functional copy of the *IRE* (*wg*^*spd-fg*^/+, Fig. 4), suggesting that TnaA is involved in the regulation of *wg* expression through this enhancer.

To further test the idea that TnaA acts on specific regulatory regions of *wg* to modulate *wg* transcription in the IR domain, we expressed *tna-Flag* on T3 leg and wing discs using the *rn-GAL4* driver^31^, and we compared the effect of TnaA-Flag on *wg* expression in both types of discs (Fig. 7B). We found that in the presence of TnaA-Flag, the Wingless level increases significantly at the wing (Fig. 7B, left), where the *IRE* and the putative D/V enhancers are active. In contrast, in the T3 leg discs, where *wg* expression is driven by the *VDE*^8^, the Wingless level is not altered or expanded (Fig. 7B**,** right), suggesting that TnaA influences specifically the *IRE* and not the *VDE*.

Then, we investigated whether TnaA and TnaA-Flag are present in the *IRE* in the chromatin of the larval wing discs. To determine this, we performed ChIP-qPCR assays of both proteins in the *wg IRE* and *VDE* enhancers (Fig. 7C). Chromatin from wing discs with wild-type TnaA or TnaA-Flag driven by *MS1096* genotypes was immunoprecipitated with anti-TnaA_TAIL_ (Sup. Fig. 1D) or anti-FLAG antibodies, respectively. The presence of TnaA in both enhancers was evaluated by qPCR amplification with the appropriate oligonucleotides (Fig. 7A). We found that both TnaA and TnaA-Flag are enriched in the *IRE* compared to their mock fractions (see “Methods”). Additionally, TnaA enrichment was not found in the *VDE* compared to its mock sample (Fig. 7C), showing that TnaA is present in the *IRE* but not in the *VDE* in the chromatin of the wing discs.

We conclude from these experiments that TnaA and TnaA-Flag are physically present in the *IRE* probably to modulate *wg* expression in the IR domain in the wing disc.

## Discussion

In this work, we found that TnaA modulates the expression of *wg* at the D/V boundary and the IR domains in the wing disc. At the D/V boundary domain, TnaA modulates *wg* expression interacting with the Notch pathway, and at the IR domain, it promotes *wg* transcription probably through its specific binding to the *wg IRE.*

Several pieces of evidence in this work indicate that TnaA is involved in the transcription of Notch target genes expressed in the D/V boundary. TnaA plays a role in the transcriptional activation of *wg* in the D/V boundary domain mediated by CSL, the effector complex of the Notch pathway. The CSL complex consists of NICD, Mam, and Su(H). According to models based on genetic and biochemical data, NICD acts as a permissive signal (e.g., *wg* transcription), alleviating the repression imposed by corepressors bound to Su(H), while in others (e.g., *ct* and *NRE-GFP* transcription), NICD acts as an instructive signal by directly recruiting transcription factors to activate gene expression^32^. In this model, overexpression of *Su(H)* reduces *ct* expression, presumably because excessive Su(H) can titrate available NICD as well as any co-repressors^18,19^. ChIP-seq analyses^33^ and studies on protein-enhancer dynamics^34,35^ showed that NICD binding has different effects on the dwell time of CSL subunits in regulatory regions and on transcriptional outcomes depending on enhancer priming and the chromatin landscape.

Our data suggest that TnaA may facilitate the formation of CSL complexes at their dedicated promoters. Loss of TnaA decreases the Wingless signal in the D/V boundary in *tna* clones and enhances the decrease in *wg* caused by mutations in Su(H) or NICD^4,5^. Conversely, TnaA-Flag increases *wg* expression and reduces *ct* and *NRE-GFP* (Fig. 3), in a behavior similar to that found in wing discs with misexpression of *Su(H)*.

Although the precise mechanism of the TnaA function remains elusive, these results fit the interpretation that TnaA is acting in the same direction as the CSL activator complex to facilitate *wg* expression in this region. Moreover, it is likely that TnaA could be involved in the regulation of the dynamics of CSL complexes or in the chromatin landscape around its target genes.

TnaA also modulates the expression of *wg* in the IR (Figs. 4, 5, 6). The loss of TnaA diminishes the Wingless signal specifically in the IR in *wg*^*spd-fg*^/+ wing discs that are haploinsufficient for the *wg* IR enhancer^10^ (Fig. 4a). In contrast, TnaA-Flag rapidly increases the Wingless signal at the IR, particularly before the *wg* IR domain is resolved in early to mid-third instar larvae. Its effect is milder after the IR formation. These results suggest that at least some regulatory regions that modulate *wg* expression in the IR are available and highly sensitive to TnaA doses throughout the development of the wing disc and may reflect the intrinsic ability of TnaA to interact with transcriptional activators and/or to find or influence chromatin changes to make its target regions available for preparation (as for enhancer priming), activation, or maintenance of *wg* expression in the IR.

TnaA is nuclear in all cells of the wing disc (this work, and^14^). However, it modulates specifically the expression of *wg* at the D/V boundary and the IR domains. This indicates that TnaA is not promiscuous and that it probably targets regulatory elements of *wg* that are functional only in specific regions within the wing disc. The complex spatial pattern of *wg* in the wing disc is regulated through multiple regulatory regions. The broad band enhancer at the notum of the wing disc and the *VDE* ventral disc enhancer are very close to each other and together with the *IRE* are accurately located^5,8^. Furthermore, enhancers that specify wings and respond to damage have recently been located close to each other^27^. In contrast, the location of the D/V boundary enhancer that is controlled by the Notch pathway^4,5^ is not known.

We found that TnaA and TnaA-Flag are in the *IRE* (Fig. 7C), influencing the expression of *wg*. To the best of our knowledge, there is not yet a compilation of which proteins are in this enhancer under wild-type conditions when the enhancer is primed, active, or inactive. Good candidates are key factors for the initiation and establishment of *wg* expression in the IR domain. These are Nubbin, Rotund, Vestigial, and Scalloped^7,36,37^. Until now, there is little evidence supporting the direct binding of any of these proteins to the *IRE* and it will be important to determine whether any of them collaborate with TnaA.

The precise location of the Notch-responsive D/V boundary enhancer remains unknown, making it difficult to test whether TnaA binds to it. However, a region located at or near the *IRE* may mediate Notch regulation of *wg*. Under conditions with ubiquitous activation of the Notch pathway, NICD and Su(H) are in a region that includes the *IRE* and an *IRE-GFP* reporter construct responds to Notch activation^33,38^. When *Su(H)* is overexpressed, there is an increase in *wg* expression in the IR^18^. This evidence indicates that there is a Notch-responsive region at or near the *IRE* that could mediate the Notch regulation of *wg* at the D/V boundary. According to this, TnaA would modulate the expression of *wg* in the IR and in the D/V boundary domains by binding to a single region that comprises the *IRE* and, at least partially, the putative D/V boundary enhancer in the wing disc. Nevertheless, our own data show that there is no change in the Notch-regulated D/V *wg* expression in animals with a reduced dose of *tna* upon *IRE* haploinsufficiency (Fig. 4). Altogether, these data leave open the possibilities that the Notch-responsive regulatory region is not affected by the deletion harbored by the *wg*^*spd-flag*^ allele or that it is located elsewhere.

We do not know whether TnaA is in the *IRE* in all cells of the wing disc or only in cells of the IR since our ChIP-qPCR experiments were carried out with chromatin from whole discs (Fig. 7C). In the wing disc, the *IRE* should only be active in the stripe of cells that form the IR (and probably in the ones that form the D/V boundary), but it is not known in which activity state the *IRE* is in other nuclei outside these *wg* expression domains.

Finally, it is interesting to discuss the role of TnaA in the function of the *wg IRE* and the D/V enhancers. TnaA is a putative SUMO E3 ligase whose targets could be protein factors recruited to the enhancer and/or histones in particular nucleosomes, histone modifiers, or chromatin remodelers surrounding it. In fact, TnaA has been implicated in the SUMOylation of subunits of the BRAHMA complex^12,14^. The BRAHMA complex has already been implicated in Notch signaling in *Drosophila* and vertebrates^39–41^.

Complexes recruited in enhancers must be very dynamic to respond to signaling at dedicated locations. The study of the mechanisms that help chromatin render a functional environment for the action of these complexes is of particular interest. SUMOylation can modify the activity, location and/or interaction of nuclear target proteins (reviewed in^11^), and TnaA has two domains that are relevant in this context^12,14^. One is the 300 aminoacidic XSPRING domain with a signature zinc finger of a kind of SUMO E3 ligases and a glutamine-rich region that can help recruit transcription-related factors that also have glutamine-rich regions^42^. Although the specific role of TnaA in *wg* transcription is not clear, one possibility is that it helps one or more regulatory factors to facilitate their exchange on enhancers such as the *wg IRE* at the wing disc.

Our data suggest that TnaA is an enhancer-specific factor of *wg*, modulating only the *wg* D/V and IR enhancer(s) of the wing imaginal disc. We do not know whether this effect is caused by the interaction of TnaA with a factor that is common to both enhancers or whether they bind to different proteins. Furthermore, if the *wg IRE* is subjected to the same kind of dynamics discovered for the CSL enhancers, TnaA may play a role in the mechanisms that determine the timing and exchange of the proteins recruited to switch the enhancer to its active or inactive forms. It remains a challenge to determine, in the tightly regulated multiple-tier network of wing disc proliferation and patterning processes, the different elements that help TnaA find, engage and exert its function at specific target genes in different wing disc domains. This will be important in understanding the role of TnaA in this context and in a genome-wide perspective.

## Methods

### Ethics statement

*Drosophila melanogaster* handling was approved by the Instituto de Biotecnología, UNAM, Bioethics Committee, Permit Number 359, which follows NOM-062 animal welfare Mexican law. No other animals were used in this study. All efforts were made to minimize animal suffering. Flies were sacrificed by CO2 euthanasia.

### Fly strains, growth, and genetic procedures

The lesions of *N*, *Su(H)*, *wg*, *tna* alleles, *lacZ* reporters for some genes, and GAL4 driver lines used in this work are, unless otherwise noted, described in Flybase^43^. The mutant alleles of *tna* used in this work are shown in Sup. Fig. 1A. Briefly, *tna*^*1*^, *tna*^*5*^ are dominant negative and null EMS-derived alleles, respectively. *tna*^*EY22929*^ is a hypomorphic *P{EPgy2}* element insertion-derived allele^44^. These three alleles are described in^13^*. tna*^*MI01482*^ is a *MiMIC* element insertion-derived allele^45^. *tna* knockdown was achieved by expressing interference RNA (RNAi) from the *tna*^*JF02536*^ line from Perrimon’s pVALIUM10-derived TRiP collection^21^, using different drivers. The efficacy of *tna* knockdown of this line (*tna*^*JF02536*^) was previously characterized^13^.

The reporter construct *NRE*-*GFP* (*Notch Responsive Element*, *NRE*) has three Grainy Head (Grh) binding sites, followed by two pairs of Su(H)-binding sites from the *Enhancer of split m8* [*E(spl)m8*] gene which is regulated by Notch as an instructive signal^20^.

The *wg*^*spd-fg*^ allele is a small deletion that removes the *IRE* region that, hence, lacks *wg* expression at the IR but the one at the D/V boundary appears to remain unaltered^10^.

Fly culture and crosses were performed according to standard procedures. Flies were raised in yeast-molasses media at 25 °C unless otherwise noted.

Notched-wing phenotypes were scored in adult animals with *tna* mutant alleles under a Notch loss-of-function genetic background. Penetrance and expressivity of the notched-wing phenotype were classified as weak or strong depending on the presence of single or several notches respectively in a wing margin (see Fig. 1B and Table 1). Adult wings were dissected in 70% ethanol, mounted onto slides in isopropanol, and immediately imaged with an Amscope UCMOS05100 camera attached to a Nikkon Eclipse E600 microscope. The statistical significance of the Notch loss-of-function phenotypes was determined using a *t*-test (P < 0.05) in animals obtained from at least three independent crosses for each genotype.

*tna*^*1*^ homozygous clones were generated by homologous recombination using *Ubx-FLP*^46^ as described in^13^. Clones expressing *tna-Flag* were generated with the FLP-out technique^47^. To remove the stop cassette in *hs-FLP, UAS-mCD8::GFP; UAS-tna-Flag/*+*; Act5C-STOP-GAL4* larvae, we induced a heat-shock in these animals at 82 h AEL in a water-bath for 15 min at 35 °C. In both cases, the wing imaginal discs were dissected from wandering third instar larvae at 96 h AEL and immunostained as described in the following section.

To induce the expression of *tna-Flag* at different stages of development, we used the Temporal and Regional Gene Expression Targeting (TARGET) system^28^. *MS1096-GAL4, UAS-GFP; UAS-tna-Flag/*+*; tub-GAL80*^*ts*^*/*+ animals were grown at 18 °C and then shifted to 29 °C at 36, 60 and 90 h AEL. Temperature changes were applied at least 12 h before and after the appearance Wingless in the pouch (48 h AEL) and IR (72 h AEL). Wing discs were dissected at around 96 h AEL and *tna-Flag* expression was corroborated by immunostaining with anti-TnaA_TAIL_ antibody (1:250, see ahead). The results were compared with the ones obtained with flies without induction (grown at 18 °C) or with full induction (grown at 29 °C).

### Characterization of epitope-tagged TnaA-Flag *Drosophila* transgenic lines

TnaA-Flag is a tagged version of TnaA_123_ from the *Iso*1 strain, which is the main nuclear isoform derived from the *tna* locus^13,14^. The FLAG epitope (DYKDDDDK) was inserted into the carboxy-termini of TnaA_123_, precisely after the last amino acid (Asp1109). The correct tagging was confirmed by DNA sequencing of the construct. The epitope-tagged TnaA version was subcloned in the p*UAS*T plasmid, where its expression is controlled by the GAL4-*UAS* system^48^. The correct molecular weight of TnaA-Flag was confirmed by Western analysis of soluble protein extracts from wing discs using the anti-TnaA_XSPRING_ antibody (Sup. Fig. 1E).

Independent transgenic *yw; UAS-tna*-*Flag* lines were obtained, and the different insertions of the transgene (*w*^+^) were mapped to different *Drosophila* chromosomes with subsequent balancing using classical genetic techniques. The TnaA-Flag protein from several transgenic lines, expressed with different strong and weak drivers, was tested for complementation of lethality caused by heteroallelic *tna* mutant alleles. It partially rescues adult viability (2% of the expected progeny), probably due to incorrect time/space dosages of the TnaA-Flag version in the whole fly. TnaA-Flag, as the wild-type form, is nuclear in imaginal discs, as corroborated by immunostaining of imaginal discs of third instar larvae with the anti-Flag antibody (Sup. Fig. 1B). TnaA-Flag binds to the same polytene bands as wild-type TnaA_123_ bands (Sup. Fig. 1C).

### TnaA antibodies and Western blot analyses

To detect TnaA in this work, we used affinity purified anti-TnaA_XSPRING_ and anti-TnaA_TAIL_ antibodies. Briefly, antibodies were raised in rabbits against different regions of the TnaA_123_ isoform encoded by the *tna-RD* transcript (Sup. Fig. 1D**)**, identified and sequenced by^49^, and reported by Flybase^49^. The anti-TnaA_XSPRING_ is described in Rosales-Vega et al.^13^, and the anti-TnaA_TAIL_ was raised against the 21-mer DVDPMEILSYLDPQPDLNTPPS peptide (aminoacids 1070–1091 of TnaA_123_) by ProSci, Inc. Both antibodies were affinity-purified from total sera. Western blot analyses of TnaA and actin in soluble protein extracts from wing discs (Sup. Fig. 1E) were performed in duplicate from two different biological replicates with soluble protein extracts from 30 wing discs of each genotype with the appropriate antibodies and were performed as specified in^13^. The membrane chemiluminescence imaging was acquired using a BioRad ChemiDoc imaging system. The raw images were not processed in any manner, and the average level of the normalized signal intensity of the indicated bands with respect to the wild-type levels (dashed line), is represented as bars with standard deviation in Sup. Fig. 1E. These proteins were detected with anti-TnaA_XSPRING_ and mouse anti-Actin (JLA20, Developmental Studies Hybridoma Bank) antibodies, used at 1:250 and 1:3000 dilutions, respectively. Secondary antibodies used were anti-rabbit HRP goat IgG (H + L) (62–6129), and anti-mouse HRP goat IgG/IgA/IgM (H + L) (A106868) (Invitrogen) used both, at 1:5000 dilutions.

### Immunostaining of polytene chromosomes and imaginal discs

To determine the colocalization between endogenous TnaA and TnaA-Flag on polytene chromosomes, we induced the expression of *tna-Flag* in the salivary glands of third instar larvae with the *Sgs3-GAL4* driver^50^ and immunostained these chromosomes (Sup. Fig. 1C). For immunostaining of polytene chromosomes, we followed the protocol of^51^, with modifications as described in^14^. Affinity purified anti-TnaA_XSPRING_ (1:50), and anti-FLAG (M2 Invitrogen) (1:100) antibodies were used in the indicated dilutions. Imaginal discs immunostaining was performed according to the protocol of^52^, with some modifications as described in^13^. To detect TnaA and TnaA-Flag, imaginal discs were immunostained with anti-TnaA_TAIL_ antibodies used at a 1:50 dilution unless otherwise specified. Other antibodies used were anti-Wg 4D4 (1:25)^53^, anti-NICD (C17.9C6) (1:200)^54^, anti-Ct 2B10 (1:50)^55^, anti-βGAL 40-1a (1:50)^56^, anti-Su(H) C-9 (Santa Cruz # sc-398453) (1:200) and anti-FLAG antibody (M2, Sigma) (1:100). Secondary antibodies anti-rabbit, anti-mouse Alexafluor 568 and anti-rat Alexafluor 594 (Invitrogen) were used for confocal microscopy. Fluorescent images of immunostained polytene chromosomes and/or imaginal discs were acquired with an Olympus Inverted FV1000, or 2P Upright FV1000 confocal microscopes with a 20X, NA 0.75 or 60X, NA 1.3 objectives. The images were processed with Fiji (ImageJ) v. 1.0, and Adobe Photoshop CS software.

### Quantification of signal intensity in confocal images

The presence of Wingless, Cut, and NRE-GFP was quantified using the plot profile tool from Fiji (ImageJ) v. 1.0. The mean grey value was used as a measure of the intensity of the signal in rectangular areas and its average value was obtained with measurements of at least six wing discs of each genotype. Statistical analyses of differences in signal intensity, were performed using Student’s *t*-test (with P-values ≤ 0.05*).

To measure the differences in *wg* expression in the genetic interaction between *tna* and *N*, we measured the intensity of the signal in ten bins of 20 µm in width around the center of the wing disc (Sup. Fig. 4A). To measure changes in the levels of Wingless, Cut, and *NRE-GFP* in *C96* > *tna-Flag* wing discs, we normalized the signal intensity values by subtracting the signal intensity in TnaA-Flag (+) cells from that in TnaA-Flag (−) cells. (Sup. Fig. 4B). To quantify changes in *wg* expression in *ptc* > *tna-Flag* wing discs, we applied the same normalization procedure as in Sup. Fig. 4. The signal intensity was quantified in the pouch, IR, and D/V (Sup. Fig. 4C).

### ChIP-qPCR analyses

Chromatin immunoprecipitation was performed on 60 wing imaginal discs from *Ore*R wandering third instar larvae per biological sample as described in^57^ with minor changes. IgA- and IgG-coupled Dynabeads (Invitrogen), were used in a 1:1 ratio for chromatin immunoprecipitation in place of protein A or G agarose/salmon sperm DNA beads. Immunoprecipitations were performed with irrelevant antibodies that do not bind nuclear proteins and serve as Mock samples (rabbit IgG, Invitrogen #02-6102, and mouse IgG, Invitrogen #02-6502), or with 5 µg of affinity purified anti-TnaA_TAIL_. Three biological samples were obtained in each case and quantitative PCR (qPCR) reactions with the appropriate oligonucleotides were performed to amplify the selected *IRE* or *VDE* regions (indicated in Fig. 7). The experiments were carried out with three replicates of each biological sample.

qPCR reactions of different *wg* regions immunoprecipitated with the anti-TnaA_TAIL_ antibody were performed as described by^58^, in a Lightcycler 480 Real-time PCR system (Roche Applied Science) using the Maxima SYBR Green/Rox qPCR Master Mix (2X) (Thermo Scientific). The *wg* regions targeted for qPCR amplification are shown in Fig. 7. Oligonucleotide sequences to amplify a 128-bp region within the *IRE* are Fwd 5′-AAAGTTATGGGCCTCCGTCT-3′ and Rev 5′- CTGGCCGAAGAGAAGTCATC-3′, and those used to amplify a 149-bp region of the *VDE*, used as a negative control, are Fwd 5′-GGACTGGAGTGGACGGATTT-3′ and Rev 5′-CCTAATTCACGCGCCAAAGT-3′. The quantification of TnaA by ChIP-qPCR on *wg IRE* in wing discs was calculated as fold enrichment over background signal and is the average of three independent biological samples with three replicates each. Statistical analyses of differential accumulation of TnaA between samples were performed using Student's *t* test, P-values ≤ 0.05.

### Supplementary Information

Supplementary Figures.

## Data Availability

Stocks and reagents are available upon request to Martha Vázquez. The authors affirm that all data necessary for confirming the conclusions of the article are present within the article, Figures, and Table.
