# Supplementary material for: TnaA, a trithorax group protein, modulates wingless expression in different regions of the Drosophila wing imaginal disc
Source: Sci Rep. 2023 Sep 13;13:15162. doi: 10.1038/s41598-023-42169-z (PMC10499800; doi:10.1038/s41598-023-42169-z)
Supplement: Supplementary file 1 — Supplementary Figures. [file 41598_2023_42169_MOESM1_ESM.docx]

Supplementary figures for

**TnaA, a trithorax group protein, modulates *wingless* expression in different regions of the *Drosophila* wing imaginal disc.**

**Marco Rosales-Vega^1^, Diana Reséndez-Pérez^2^, Mario Zurita^1^, and Martha Vázquez^1,^***

^1^Departamento de Genética del Desarrollo y Fisiología Molecular, Instituto de Biotecnología, Universidad Nacional Autónoma de México, Cuernavaca 62210, Morelos, México.

^2^Departamento de Inmunología y Virología, Facultad de Ciencias Biológicas, Universidad Autónoma de Nuevo León, San Nicolás de los Garza, Nuevo León, México.

* Author of correspondence (e-mail: martha.vazquez@ibt.unam.mx)

**This file includes**

Supplementary Figures 1-5

**
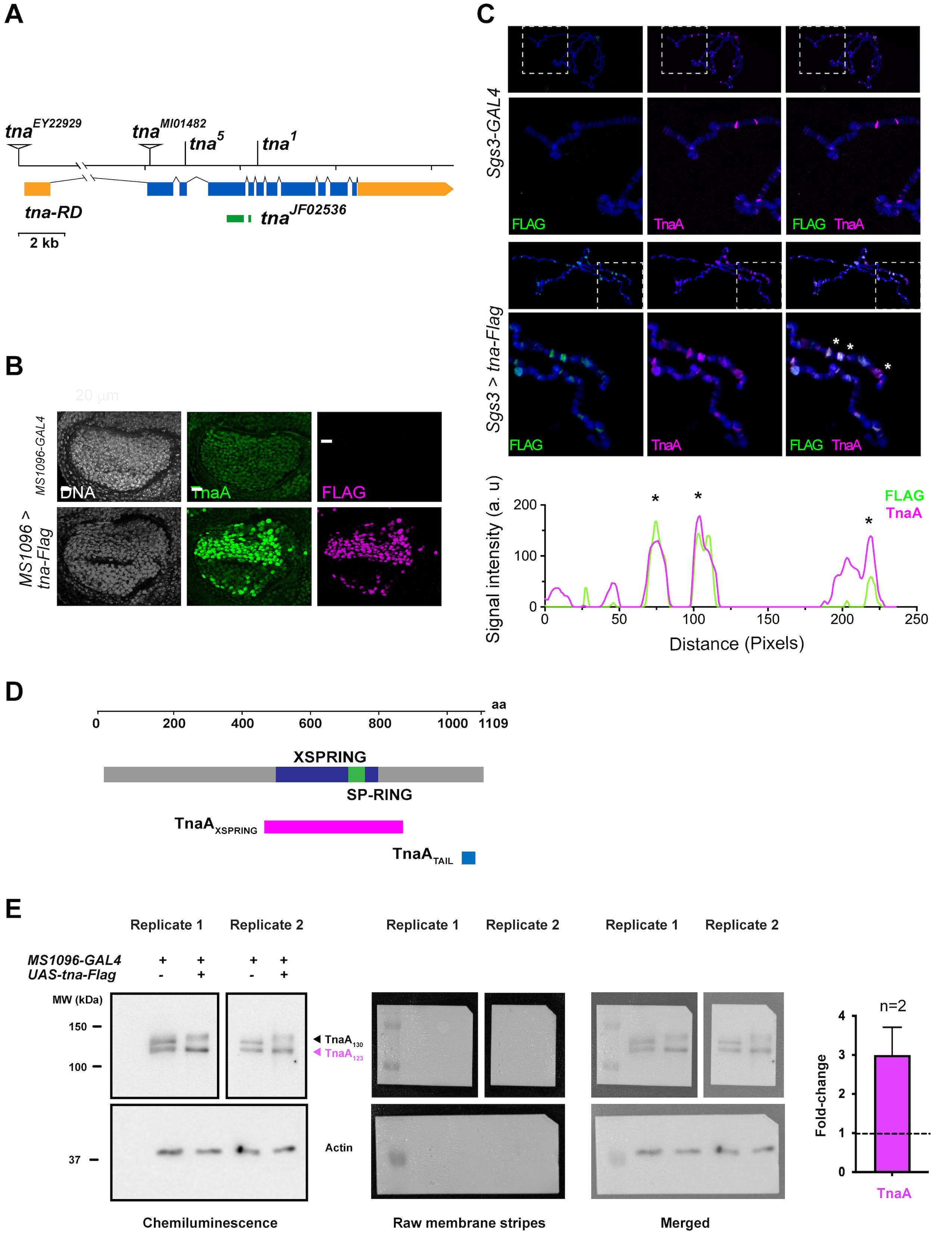
**

**Sup. Fig. 1.** *tna* mutant alleles, antibodies, and expression of *tna-Flag* in imaginal disc cells. (**A**) *tna* genomic region of the *tna-RD* transcript, (untranslated and translated exons in yellow and blue, respectively), indicating the lesions (triangle for insertion and vertical black lines for point mutations) and the *tna^JF02536^* RNAi (target region, green) used in this work. (**B**) Endogenous TnaA and TnaA-Flag localize to the nuclei of imaginal disc cells. Immunostaining of TnaA (anti-TnaA_TAIL_ antibody, green) and TnaA-Flag (anti-FLAG antibody, magenta) driven by *MS1096-GAL4*, compared to *MS1096-GAL4* haltere discs (upper panel). (**C**) TnaA and TnaA-Flag colocalize in polytene chromosome bands. The endogenous TnaA signal detected with the anti-TnaA_XSPRING_ antibody (magenta) is shown in *Sgs3-GAL4* polytene chromosomes (*Sgs3-GAL4* panels, amplified region indicated by a dashed square). The colocalization of TnaA~~,~~ and TnaA-Flag (FLAG antibody, green) driven by *Sgs3-GAL4* is shown in *Sgs3*>*tna-Flag* panels. To assess the colocalization of bands detected with anti-TnaA_XSPRING_ and anti-FLAG antibodies, we traced the signal intensity along a line in a chromosomal region (dashed square, *Sgs3*>*tna-Flag* panels) with the ImageJ plot profile tool and represented the data in a graph (bottom panel). (**D**) TnaA_123_ protein (grey, 1109 residues) indicating the XSPRING (purple) and SP-RING (green). The regions targeted by the polyclonal antibodies are shown, TnaA_XSPRING_ (pink) and TnaA_TAIL_ (blue). (**E**) Western blot analysis of *MS1096>tna-Flag* wing discs compared to control *MS1096* wing discs. To quantify the changes in TnaA between the *MS1096* and *MS1096>tna-Flag* wing discs, the full-length blot membrane with samples from two independent biological replicas, was cut into two membrane stripes to immunodetect TnaA with the anti-TnaA_XSPRING_ (upper panels), and one stripe to immunodetect actin as a loading control from replicas 1 and 2 (lower panel). Each set of corresponding TnaA and actin images shown in replica 1 and 2, come from the same gel lanes and they were processed in parallel. After immunodetection, chemiluminescent images were acquired with the ChemiDoc imaging system (BioRad). Chemiluminescence images (left), raw membrane stripes (center) and the merged images of both replicas (right) are shown. The experiment was performed by duplicate, showing at least a three-fold increase in TnaA-Flag levels (magenta bar).


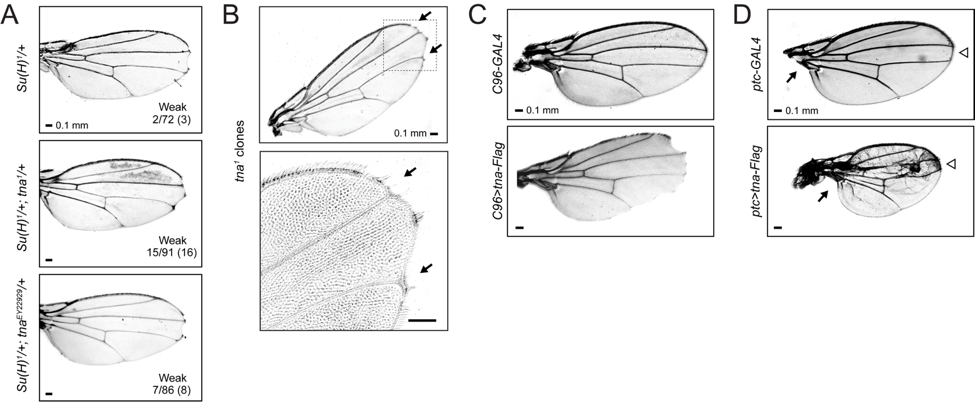


**Sup. Fig. 2.** Adult wings derived from experiments in this study. (**A**) Genetic interaction of *tna* with *Su(H).* *Su(H)^1^* wings alone or in combination with *tna* alleles (*tna^1^* and *tna^EY22929^*). *tna^1^* and *tna^EY22929^* wings do not have notching, while *Su(H)*/+; *tna/+* transheterozygotes have notched wings. The penetrance and expressivity of the notched-wings phenotype are indicated in Table 1. (**B**) Adult wings with notches from animals in which *tna^1^* clones of the wings discs were induced. The lower panel shows an amplification of the observed notches. (**C**) The expression of *tna-Flag* driven by *C96-GAL4* (lower) causes strong notching in adult wings compared to the *C96-GAL4* control (upper); (**D**) *ptc-GAL4* (upper) and *ptc>tna-Flag* adult wings (lower), indicating the hinge (arrow) and the D/V boundary (empty arrowhead).


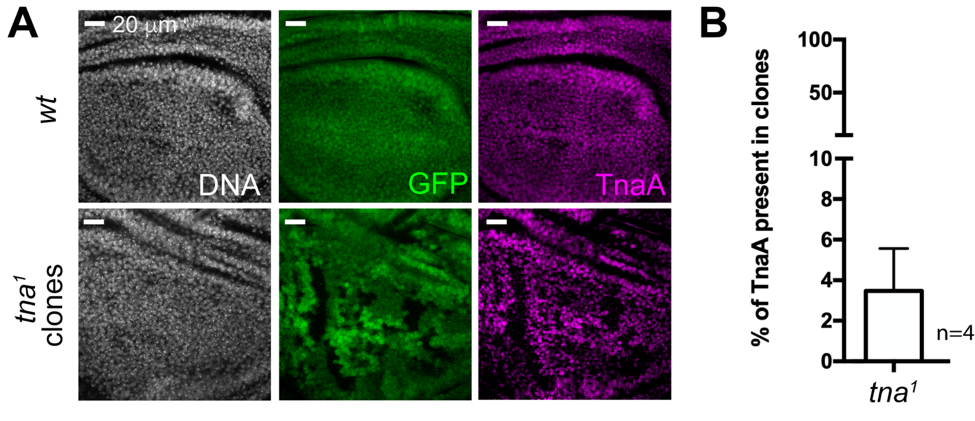


**Sup. Fig. 3.** TnaA signal in *tna^1^* clones. (**A**) Mitotic clones (non-GFP cells) were induced with *Ubx-FLP* in wing imaginal discs. DNA, TnaA (magenta), and GFP (green) are shown. (**B**) *tna^1^* clones show only 5% of the wild-type TnaA signal when immunostained with the anti-TnaA_TAIL_ antibody.


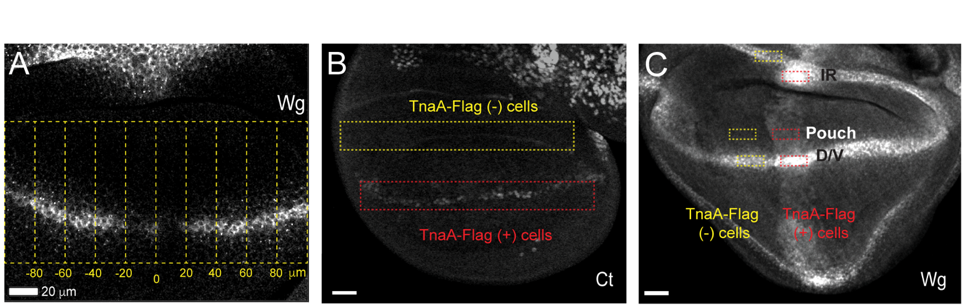


**Sup. Fig. 4.** Quantification of Wingless and Cut signal intensities in immunostainings of wing imaginal discs. (**A**) Example of a *N^55e11^*/+; *tna^1^*/+ wing disc divided into 10 regions (20 µm in width) along the *wg* D/V boundary domain. These regions allow quantification of the Wingless signal around the center of the D/V boundary compared to the Wingless signal found in a similar region in a wild-type disc. (**B**) Example of quantification of Cut signal intensity in a *C96>tna-Flag* wing disc. To make an accurate quantification of the signal, we normalized the Cut signal intensity by subtracting the signal in the TnaA-Flag (-) cells from the signal obtained in TnaA-Flag (+) cells. (**C**) Example of quantification of the intensity of the Wingless signal in a *ptc>tna-Flag* wing disc*.* Signal intensity was normalized as in (**B**) for each of the D/V, IR, and pouch regions.


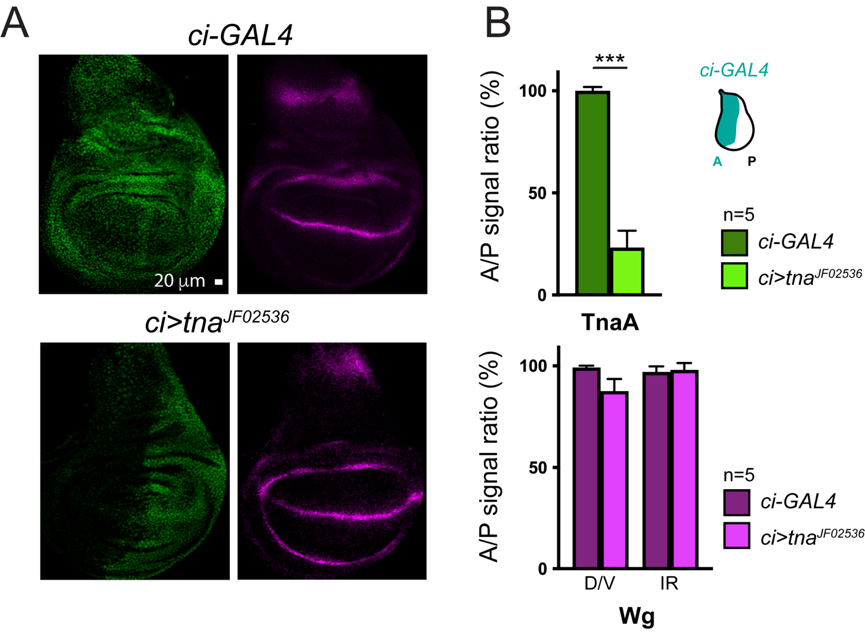


**Sup. Fig. 5.** Effectiveness of TnaA knockdown and its effect on Wingless signal in the D/V and IR regions. (**A**) TnaA (green) was knockdowned in the anterior compartment by the expression of a RNAi from the *tna^JF25036^* allele driven by the *ci-GAL4*. Wingless signal (magenta) was analyzed in this genotype. (**B**) Quantification of the A/P signal ratio of TnaA (upper panel) and Wingless (lower panel). Note that on average TnaA is reduced to 20% of the wild type signal compared to 5% of the signal observed in *tna^1^* clones from Sup. Fig. 3. Student’s *t*-test was performed for A/P signal ratios (P<0.001^***^).
